# A retrospective chart review study of symptom onset, diagnosis, comorbidities, and treatment in patients with binge eating disorder in Canadian clinical practice

**DOI:** 10.1007/s40519-020-01026-y

**Published:** 2020-10-10

**Authors:** Simerpal K. Gill, Allan S. Kaplan

**Affiliations:** 1 Bay Adelaide Centre, Medical Affairs, Takeda Canada Inc, 22 Adelaide Street West, Suite 3800, Toronto, ON M5H 4E3 Canada; 2 Department of Psychiatry, University of Toronto, Toronto, ON Canada

**Keywords:** Adult, Binge eating disorder, Diagnosis, Symptom onset, Treatment

## Abstract

**Purpose:**

In the Canadian healthcare setting, there is limited understanding of the pathways to diagnosis and treatment for patients with binge eating disorder (BED).

**Methods:**

This retrospective chart review examined the clinical characteristics, diagnostic pathways, and treatment history of adult patients diagnosed with BED.

**Results:**

Overall, 202 charts from 57 healthcare providers (HCPs) were reviewed. Most patients were women (69%) and white (78%). Mean ± SD patient age was 37 ± 12.1 years. Comorbidities identified in > 20% of patients included obesity (50%), anxiety (49%), depression and/or major depressive disorder (46%), and dyslipidemia (26%). Discussions regarding a diagnosis of BED were typically initiated more often by HCPs than patients. Most patients (64%) received a diagnosis of BED ≥ 3 years after symptom onset. A numerically greater percentage of patients received (past or current) nonpharmacotherapy than pharmacotherapy (84% vs. 67%). The mean ± SD number of binge eating episodes/week numerically decreased from pretreatment to follow-up with lisdexamfetamine (5.4 ± 2.8 vs. 1.7 ± 1.2), off-label pharmacotherapy (4.7 ± 3.9 vs. 2.0 ± 1.13), and nonpharmacotherapy (6.3 ± 4.8 vs. 3.5 ±  6.0) Across pharmacotherapies and nonpharmacotherapies, most patients reported improvement in symptoms of BED (84–97%) and in overall well-being (80–96%).

**Conclusions:**

These findings highlight the importance of timely diagnosis and treatment of BED. Although HCPs are initiating discussions about BED, earlier identification of BED symptoms is required. Furthermore, these data indicate that pharmacologic and nonpharmacologic treatment for BED is associated with decreased binge eating and improvements in overall well-being.

**Level of evidence:**

IV, chart review.

## Introduction

Binge eating disorder (BED) is characterized by the recurrent consumption of an amount of food within a discrete period that is larger than what most people would eat, by a lack of control, and by marked distress over binge eating (BE) [1]. Studies suggest that BED has a neurobiologic basis [2], with altered function in corticostriatal dopaminergic systems most likely playing a key role in the increased impulsivity and compulsivity, decreased reward sensitivity, and attentional biases toward food associated with BED [3].

The prevalence of BED based on *Diagnostic and Statistical Manual of Mental Disorders, Fifth Edition* (*DSM-5*) criteria has been examined in several studies [4–7]. In a US population-based survey, the lifetime prevalence of BED in adults was 2.03% [4]. In a UK-based study, BED point prevalence in a multi-ethnic population was 3.6% [5]. In a Canadian population-based survey, the lifetime prevalence of BED was 3.19% [7]. Epidemiologic studies indicate that the prevalence of BED is generally higher than that of anorexia nervosa (AN) and bulimia nervosa (BN) [8–10].

Psychiatric and medical comorbidities are frequently observed in individuals with BED [8, 10, 11]. In a study that used data from the National Epidemiologic Survey on Alcohol and Related Conditions-III, 93.8% of respondents meeting *DSM-5* BED criteria also met criteria for ≥ 1 additional lifetime psychiatric disorder; the lifetime prevalence of having any mood disorder was 69.9% and any anxiety disorder was 59.0% [10]. In the same study, the most frequent metabolic disorders included hypertension (31.2%), hypercholesterolemia (27.2%), and diabetes (13.6%) [10]. In a large epidemiologic survey of 14 countries, a lifetime diagnosis of BED was found to be significantly associated with an increased risk of having psychiatric disorders, hypertension, and diabetes [8].

Untreated or undertreated BED poses health and economic burdens [12–16]. In a US-based survey of working adults, respondents meeting BED diagnostic criteria had significantly higher percentages of absenteeism, presenteeism, and work productivity loss than those not meeting BED diagnostic criteria [16]. Furthermore, another US-based survey found that individuals meeting BED diagnostic criteria had higher healthcare resource utilization and costs compared with individuals not meeting BED diagnostic criteria [12]. Given the evidence indicating that individuals with BED have a higher incidence of dyslipidemia, hypertension, and type 2 diabetes [17], the costs associated with those comorbid conditions could compound the existing economic burden of BED. These findings emphasize the need to diagnose and treat BED in a timely manner to improve health outcomes and mitigate the costs of BED, which may be achieved by early identification and management in the general practice setting [18].

Treatment for BED includes nonpharmacotherapies and pharmacotherapies. Nonpharmacotherapies include cognitive behavioral therapy (CBT), interpersonal therapy, and behavioral weight loss therapy [19, 20]. Lisdexamfetamine dimesylate (LDX), which is approved for use in multiple countries, including Canada, is the only approved pharmacotherapy for patients with BED [21, 22]. The Health Canada approved LDX starting dose for moderate to severe BED treatment is 30 mg once daily; the dose can be increased as tolerated to 70 mg once daily [22]. Although other pharmacotherapies (e.g., antidepressants, anticonvulsants, antiobesity agents) have been used to manage BED, their use for BED is not approved by regulatory agencies, and limited data exist regarding their efficacy and safety [23, 24].

Currently, there is a poor understanding of the path from BED symptom onset to diagnosis and treatment in the Canadian healthcare setting. The Canadian primary healthcare system varies from province to province, but patients are generally required to see their primary care physician to be referred to a specialist (i.e., a psychiatrist). There are no restrictions on primary care physicians prescribing psychostimulants as there are in other jurisdictions. It is not uncommon for primary care physicians, as well as psychiatrists, to diagnose and treat patients diagnosed with BED with pharmacotherapy. Understanding the pathway taken by patients with BED is essential to ensure a timely diagnosis and to optimize treatment and subsequent outcomes. The objectives of this retrospective chart review were to describe symptom onset, diagnosis, comorbidities, and BED treatments and outcomes in patients diagnosed with BED symptoms in the Canadian clinical setting.

## Methods

### Study design

This retrospective chart review assessed Canadian adults diagnosed with BED by a community- or hospital-based healthcare provider (HCP) who was either a general practitioner (GP) or psychiatrist. The study protocol and case report forms (CRFs) were reviewed and approved by a central institutional review board (IRB), which also provided ethics approval (Advarra IRB; Columbia, MD). Sites were excluded if they required local IRB approval or if they did not have permission to provide deidentified patient data. When applicable, patient consent was obtained. Patients included in this chart review were men and women aged ≥ 18 years at the time of BED diagnosis. Eligible patients had an HCP visit between July 1, 2017, and August 31, 2018; a current BED diagnosis; ≥ 1 follow-up visit after treatment initiation (if treated); and no other current documented eating disorder.

Healthcare provider respondents were recruited from 57 Canadian sites and were identified from a research database of HCPs who had previously participated in BED-related research. Each site received access to secure online CRFs onto which data were entered (in English or French) by the site investigator or a designated staff member between October 9, 2018, and December 10, 2018. Each patient was assigned a randomly generated and encrypted study identification number; no identifying information was recorded.

### Endpoints

Participating HCPs were requested to provide data extracted from a maximum of 15 medical charts. Respondents were asked questions about patient sociodemographic characteristics, lifetime diagnoses of psychiatric and medical comorbidities, and BED diagnostic history (first consultation, who initiated the discussion about BED, and what prompted the discussion of BED [e.g., suspected symptoms], co-occurring psychiatric or medical disorders)], treatment histories) [nonpharmacotherapy and pharmacotherapy [on- and off-label)], and treatment effects at follow-up relative to diagnosis. Treatment discontinuation was assessed based on whether the patients’ chart indicated the patient was “currently taking” or “no longer taking” treatment. Most data were transcribed verbatim from patients’ charts to the standardized CRF; some data required the HCP to provide a clinical impression of patients’ status and response to treatment (i.e., improved, unchanged, worsened) based on review of the patient chart.

Data were collected from patients who had visited the HCP in the past 12 months (between July 1, 2017, and August 31, 2018) and the visit was at least the second BED consultation. To minimize recall bias, HCPs entered data into the CRF to ensure that patient charts were accurately transcribed. As this study was a chart review, there was no systematic follow-up. Follow-up was based on individual patient needs, was not dictated by the study, and varied from person to person in a manner reflective of real clinical practice.

### Data presentation

MD Analytics (Vancouver, BC, Canada) collated the CRFs and analyzed the data. Descriptive statistics are reported for all measures. To assess clinical outcomes, the number of BE episodes/week at diagnosis and follow-up was examined (if available in the patient’s chart). Additionally, patients were categorized as improved, unchanged, or worsened regarding BED symptoms and overall well-being.

## Results

### Patient disposition and demographics

A total of 4729 invitations were sent to HCPs (GPs, *n* = 3823; psychiatrists, *n* = 906) requesting participation. In response, 175 HCPs (GPs, *n* = 114; psychiatrists, *n* = 61) visited the website and 57 (GPs, *n* = 32; psychiatrists, *n* = 25) participated. A total of 202 patient charts (125 from GPs, 77 from psychiatrists) were reviewed.

Most patients were women, white, and aged 18–54 years. The highest percentage of participants had a body mass index in the 30.0–39.9 kg/m^2^ range (Table 1). Approximately one-third of patients were unemployed. The mean ± SD number of BE episodes/week was 5.4 ± 3.8 (median, 5 years) at the time of diagnosis. This distribution of BE episodes/week at diagnosis is reported in Table 1.

**Table 1:** Patient Characteristics

	Patients (*N* = 202)
Sex, *n* (%)	
Women	139 (69)
Age, y	
Mean ± SD	37 ± 12.1
Category, *n* (%)	
18‒34	91 (45)
35‒54	91 (45)
≥ 55	20 (10)
Race/ethnicity, *n* (%)^a^	
White/Caucasian	158 (78)
Black	10 (5)
South Asian	8 (4)
Latin American	6 (3)
Aboriginal/First Nations	6 (3)
Other	18 (9)
Region, *n* (%)	
Ontario	94 (47)
Quebec	52 (26)
West	49 (24)
Atlantic	7 (3)
Employment status, *n* (%)	
Employed	135 (67)
Body mass index, kg/m^2b^	
Mean ± SD	30.9 ± 7.5
Category, *n* (%)	
≤ 24.9	46 (23)
25.0‒29.9	63 (31)
30.0‒39.9	75 (37)
≥ 40.0	18 (9)
BE episode/week at diagnosis, *n* (%)^c^	
1–2	28 (16)
3–5	78 (45)
6–10	57 (33)
≥ 11	11 (6)

*BE* binge eating

^a^Multiple options could be selected on patient charts

^b^*n* = 199

^c^*n* = 174

### Comorbidities

**Fig. 1:**
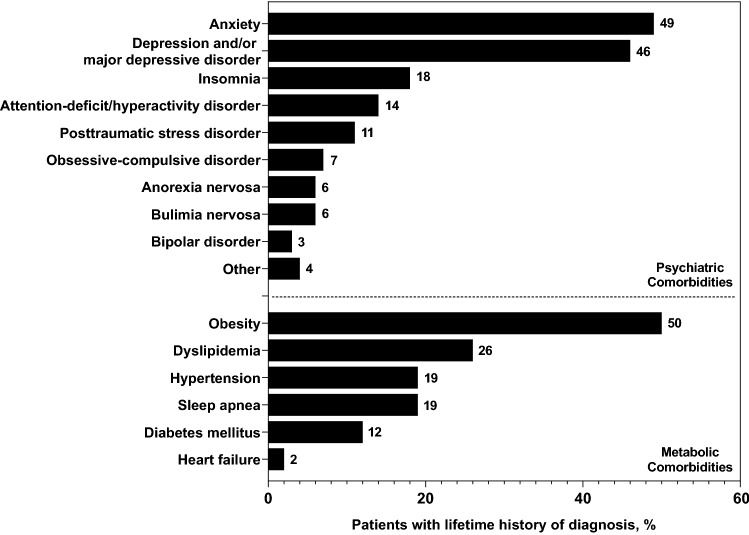
Patients with a lifetime history of any diagnosed comorbidity. ^a^ ^a^* N* = 202

Psychiatric comorbidity diagnoses (any lifetime diagnosis) observed in ≥ 5% of patients were anxiety, depression and/or major depressive disorder, insomnia, attention-deficit/hyperactivity disorder, posttraumatic stress disorder, obsessive–compulsive disorder, BN, and AN (Fig. 1). Metabolic comorbidity diagnoses (any lifetime diagnosis) observed in ≥ 5% of patients were obesity, dyslipidemia, hypertension, sleep apnea, and diabetes (Fig. 1). Only 16% (32/202) and 37% (75/202) of patients, respectively, did not have a lifetime history of a diagnosed psychiatric or metabolic comorbidity. Eighty-two percent of patients (166/202) did not have any history of a previous eating disorder diagnosis.

### BED diagnostic pathway and clinical presentation

Descriptive review of the data collected indicated that discussions about BED were initiated more often by HCPs (GPs, 65% [73/112]; psychiatrists, 80% [56/70]) than patients (GP patients, 31% [35/112]; psychiatrist patients, 20% [14/70]). The BED diagnosis was most often established by the HCP (90% [112/125] of GP patients, 91% [70/77] of psychiatrist patients). The first BED consultation was from the GP in 75% of GP patients (94/125) and from the psychiatrist in 18% of psychiatrist patients (14/77). Referral from a GP occurred in 12% of GP patients (15/125) and 69% of psychiatrist patients (53/77). Small percentages of patients were referred by a psychiatrist to a GP (6% [8/125]) or to another psychiatrist (8% [6/77]) or were diagnosed by another type of HCP (patients of GP, 6% [8/125]; patients of psychiatrist, 5% [4/77]).

**Fig. 2:**
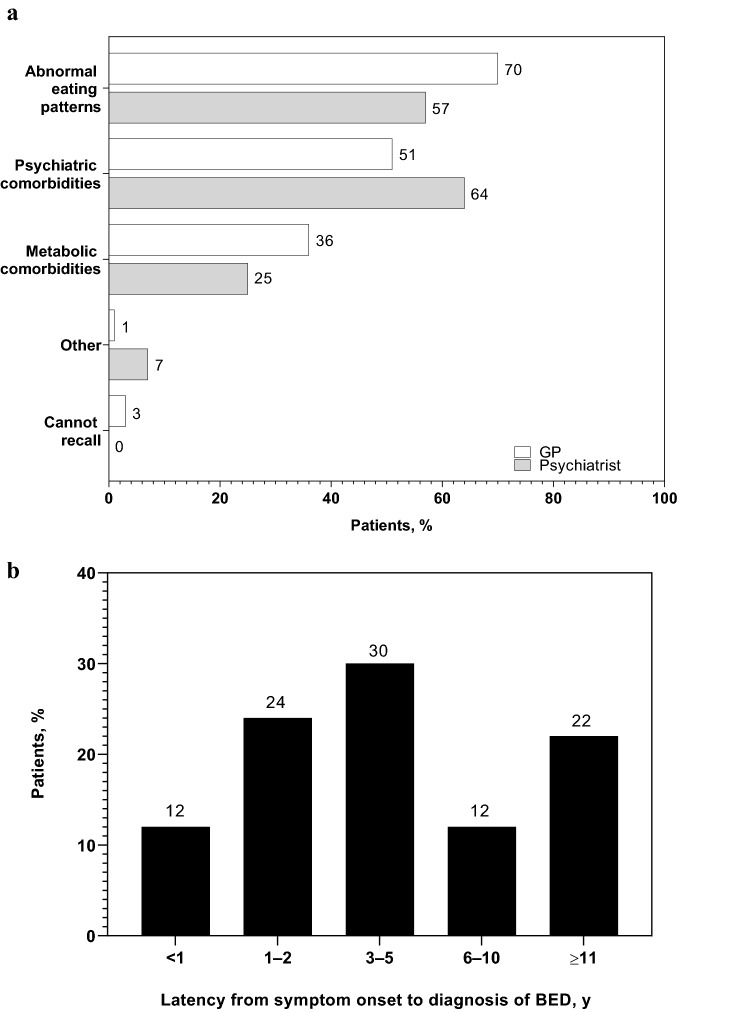
**a** Diagnostic indicators of BED.^a^**b** Latency from symptom onset to BED diagnosis.^b^ *BED* binge eating disorder; *GP* general practitioner. ^a^*n* = 73 for patients of GPs; *n* = 56 for patients of psychiatrists. ^b^*n* = 135

The most frequently observed BED diagnostic indicators were abnormal eating behaviors in GP patients and psychiatric comorbidities in psychiatrist patients (Fig. 2a). The mean ± SD latency from BED symptom onset to diagnosis was 6.4 ± 7.5 years (median, 3 years). The most frequent latency range from symptom onset to diagnosis was 3–5 years (Fig. 2b).

### Treatment

A numerically greater percentage of patients used nonpharmacotherapy than pharmacotherapy (Table 2), with nutritional counseling being the most frequently used nonpharmacotherapy (past or current) and LDX the most frequently used pharmacotherapy. Regarding pharmacotherapies, 79% of patients being treated with LDX used dosages ≥ 50 mg/day and 61% of patients being treated with bupropion used dosages of 300 mg/day.

**Table 2:** Past and Current Nonpharmacotherapies and Pharmacotherapies for BED

Therapies (*N* = 202)	Total^a^	Past treatment^a^	Current treatment^a^	Current treatment duration, years^b^			
				≤ 3	4‒8	> 8	Unknown
Nonpharmacotherapy, *n* (%)							
Any nonpharmacotherapy	169 (84)	30 (15)	139 (69)	ND	ND	ND	ND
Nutritional counseling	123 (61)	38 (19)	85 (42)	43 (51)	16 (19)	8 (9)	18 (21)
Weight loss management	103 (51)	39 (20)	64 (31)	32 (50)	11 (17)	8 (13)	13 (20)
Psychotherapy	98 (49)	26 (13)	72 (36)	46 (64)	15 (21)	6 (8)	5 (7)
Herbal preparations	30 (15)	27 (14)	3 (2)	3 (100)	0	0	0
Bariatric surgery	10 (5)	4 (2)	6 (3)	4 (66)	1 (17)	1 (17)	0
Pharmacotherapy, *n* (%)							
Any pharmacotherapy	135 (67)	22 (11)	113 (56)	ND	ND	ND	ND
LDX	77 (38)	4 (2)	73 (38)	68 (93)	5 (7)	0	0
Bupropion	34 (17)	16 (8)	18 (9)	10 (56)	5 (28)	2 (11)	1 (5)
Citalopram	18 (9)	11 (5)	7 (3)	3 (43)	2 (29)	1 (14)	1 (14)
Fluoxetine	18 (9)	11 (5)	7 (3)	5 (71)	0	2 (29)	0
Escitalopram	16 (8)	8 (4)	9 (4)	7 (78)	0	1 (11)	1 (11)
Sertraline	12 (6)	5 (2)	7 (3)	4 (58)	1 (14)	1 (14)	1 (14)
Venlafaxine	8 (4)	4 (2)	4 (2)	2 (50)	1 (25)	0	1 (25)
Duloxetine	6 (3)	3 (1)	3 (1)	2 (67)	1 (33)	0	0
Bupropion/naltrexone	6 (3)	4 (2)	2 (1)	2 (100)	0	0	0
Paroxetine	4 (2)	1 (< 1)	3 (1)	0	0	1 (33)	2 (67)
Desvenlafaxine	4 (2)	4 (2)	1 (< 1)	1 (100)	0	0	0

*BED* binge eating disorder; *LDX* lisdexamfetamine dimesylate; *ND* not determined

^a^Percentage based on total sample size (*N* = 202)

^b^Percentage based on sample size for current treatment

**Fig. 3:**
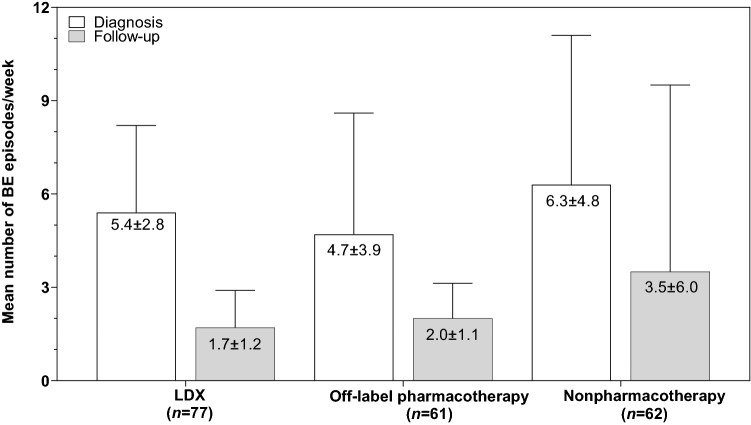
Number of BE episodes/week. Error bars represent standard deviations. *BE* binge eating; *LDX* lisdexamfetamine dimesylate

Based on treatment duration data (Table 2), high percentages of patients discontinued treatment after 3 years. The most frequently discontinued therapies were herbal preparations (90% [27/30]), desvenlafaxine (75% [3/4]), fluoxetine (61% [11/18]), bupropion/naltrexone combination (60% [3/5]), and citalopram (56% [10/18]). The least frequently discontinued therapy was LDX (5% [4/77]). Herbal preparations (85% [23/27]), bariatric surgery (67% [2/3]), and weight loss management (58% [22/38]) were the nonpharmacotherapies most frequently discontinued because of lack of efficacy; weight loss management (61% [23/38]) and diet/nutritional counselling (44% [17/39]) were discontinued because of lack of adherence. For pharmacotherapy, adverse effects were the most frequent reasons for discontinuation for bupropion/naltrexone combination (100% [3/3]), LDX (75% [3/4]) and sertraline (60% [3/5]). In contrast, lack of efficacy was recorded as a reason for discontinuation more frequently for fluoxetine (82% [9/11]), citalopram (80% [8/10]), bupropion (69% [11/16]), and venlafaxine (67% [2/3]). Numerical reductions in BE episodes/week were observed from diagnosis to follow-up with nonpharmacotherapy and pharmacotherapy (Fig. 3), with the magnitude of the treatment difference from diagnosis to follow-up being numerically larger with LDX than any other therapy for which data were available. Across therapies, the greatest percentages of patients had 1–2 BE episodes/week at follow-up (Table 3). With nonpharmacotherapy and pharmacotherapy, numerically greater percentages of patients were improved regarding BED symptoms and overall well-being at follow-up than those who were unchanged or worsened (Table 4).

**Table 3:** Distribution of BE Episodes/Week at Diagnosis and Follow-up

	LDX (*n* = 77)		Off-label pharmacotherapy (*n* = 61)		Nonpharmacotherapy (*n* = 62)	
	Diagnosis	Follow-up	Diagnosis	Follow-up	Diagnosis	Follow-up
BE episodes/week, *n* (%)						
1–2	11 (14)	61 (79)	10 (16)	36 (59)	5 (8)	28 (45)
3–5	27 (35)	8 (10)	30 (49)	12 (20)	22 (35)	10 (16)
6–10	33 (43)	1 (1)	8 (13)	0	16 (26)	4 (6)
≥ 11	3 (4)	0	2 (3)	0	5 (8)	2 (3)
Undetermined	3 (4)	7 (9)	11 (18)	13 (21)	14 (23)	18 (29)

*BE* binge eating; *LDX* lisdexamfetamine dimesylate

**Table 4:** Clinical Impressions of Current Treatment Effects for Nonpharmacotherapies and Pharmacotherapies^a^

	Reduced binge episodes^b^	BED symptoms^b^			Overall well-being^b^		
		Improved	Unchanged	Worsened	Improved	Unchanged	Worsened
Nonpharmacologic therapy, *n* (%)^c^							
Nutritional counseling	72 (85)	71 (84)	11 (13)	3 (3)	68 (80)	16 (19)	1 (1)
Weight loss management	55 (86)	55 (86)	7 (11)	2 (3)	55 (86)	8 (13)	1 (1)
Psychotherapy	66 (92)	65 (90)	6 (8)	1 (2)	64 (89)	8 (11)	0
Pharmacologic therapy, *n* (%)							
LDX	71 (97)	71 (97)	2 (3)	0	70 (96)	3 (4)	0
Bupropion	17 (94)	16 (89)	2 (11)	0	16 (89)	2 (11)	0

*BED* binge eating disorder; *LDX* lisdexamfetamine dimesylate

^a^Based on review of the patient chart

^b^Percentage based on sample size for current treatment (nutritional counseling, *n* = 85; weight loss management, *n* = 64; psychotherapy, *n* = 72; LDX, *n* = 73; bupropion, *n* = 18)

^c^Therapies with insufficient sample size were excluded from analysis

## Discussion

This retrospective chart review describes symptom onset, diagnosis, comorbidities, BED treatments, and treatment outcomes in patients diagnosed with BED symptoms in the Canadian clinical setting. The BED diagnostic pathway was characterized by discussions typically being initiated by HCPs more often than by patients, and with initial consultations typically being conducted more often by GPs than psychiatrists. The most frequently reported BED diagnostic indicators were abnormal eating patterns and psychiatric comorbidities.

Although psychiatrists were typically more likely to initiate BED discussions than GPs, the first consultation about BED tended to occur more often with GPs than psychiatrists, and a high percentage of psychiatrist patients were referred from GPs. This indicates that GPs are important points of first contact for patients with BED. However, patient factors such as lack of symptom recognition by the patient, unwillingness of the patient to discuss symptoms due to shame or stigma [25, 26], or patients seeking help for weight loss rather than disordered eating, combined with lack of comfort or knowledge regarding eating disorders among GPs may contribute to delays in BED diagnosis [26–29]. In one study, 42% of US physicians from family practice, internal medicine, or obstetrics/gynecology reported never having assessed their patients for BED [27]. In another, only 33% of US women with eating disorder symptoms (AN, BN, or BED) reported that their primary care practitioner/HCP asked them about problematic eating [29]. A survey of primary care practice physicians reported that 77% of physicians who reported familiarity with BED did not know the diagnostic criteria for BED, with 35% not recognizing that the absence of compensatory behaviors was an essential diagnostic criterion [28]. Even in psychiatric practices, discrepancies in patient and provider perceptions of BED can present barriers to diagnosis and treatment, as an analysis of provider-patient conversations indicated that psychiatrists focused on different aspects of BED-related symptoms than patients [26].

Most patients experienced BED symptoms for > 1 year before being diagnosed. If related to a BED knowledge gap, this finding is important because a study of primary care physicians reported that physicians with greater knowledge of eating disorders were more likely to suggest a follow-up appointment [30], which could facilitate a more timely diagnosis. The delay in BED diagnosis observed in this study is critically important because early recognition and treatment could help lower the overall socioeconomic burden of BED.

Based on physician reporting of current comorbidities, the most frequently observed comorbidities included anxiety, depression and/or major depressive disorder, obesity, and dyslipidemia. These findings are consistent with those of previously published reports indicating that patients with BED exhibit an increased frequency and risk of psychiatric [8–11] and medical comorbidities [10, 31]. For example, Udo and Grilo reported that mood and anxiety disorders were among the most prevalent psychiatric comorbidities in US patients diagnosed with BED, with the prevalence of hypertension (31.2%), high cholesterol (27.2%), high triglycerides (14.5%), and diabetes (13.6%) also being relatively high [10]. However, it should be noted that the specific criteria used to diagnose patient comorbidities in the current report are unknown.

In this study population, a numerically larger percentage of patients received nonpharmacotherapy than pharmacotherapy. Nutritional counseling and weight loss management were the most frequently used nonpharmacologic therapies. It was surprising that the use of CBT was not reported in any of the patients included in this chart review given that its use in BED is recommended in treatment guidelines [20, 32, 33]. A 2012 Canadian report outlining therapeutic approaches for BED recommended a multidisciplinary approach that incorporates CBT, dialectical behavioral therapy, motivational interviewing, and mindfulness [32]. This recommendation is consistent with guidelines of the American Psychiatric Association and the National Institute for Health and Care Excellence of the United Kingdom, which also recommend that some form of CBT be incorporated into BED treatment plans [20, 33].

The more frequent use of nonpharmacotherapy in these patients is understandable because a pharmacotherapy was not approved for BED in Canada until the approval of LDX in October 2016. In patients treated with the two most frequently used pharmacotherapies—LDX followed by the antidepressant bupropion—most patients used dosages within the recommended ranges (79% of patients used 50‒70 mg/day LDX and 61% of patients used 300 mg/day bupropion). This treatment profile is not surprising because LDX is the only approved pharmacotherapy for BED in Canada [22] and antidepressants have frequently been examined as potential treatments for BED [24]. It is important to note, however, that discontinuation rates for antidepressants were as high as 75% and were most often due to lack of efficacy. In contrast, LDX was the least discontinued therapy.

Regardless of therapy type (nonpharmacotherapy or pharmacotherapy), associations were observed between treatment and reductions in BE and BE symptoms and improved overall well-being. Although statistical analyses were not conducted, the magnitude of improvement across the aforementioned measures tended to be greater with LDX than with other therapies. The observation of improved outcomes with LDX in this study is consistent with evidence from the pivotal clinical studies that supported its approval for the treatment of BED in Canada [22, 34, 35]. The observed improvement with nonpharmacotherapy is also consistent with expectations based on current treatment guidelines [32, 33].

Evidence for a substantial level of treatment discontinuation within 3 years was observed, with few patients having a current treatment duration of > 3 years. The most frequently reported reasons for discontinuation of pharmacotherapy were lack of efficacy and adverse effects. For nonpharmacotherapy, it is important to consider that treatment is generally time-limited, with the defined number of sessions varying across therapies [36, 37]. As such, discontinuation of nonpharmacotherapy in this study may be related to the end of treatment. Together, these findings suggest that HCPs should consider various factors (e.g., efficacy, comorbidities, costs, and patient willingness to be treated, time to symptom improvement) that may affect treatment adherence when discussing treatment options with patients.

These results should be interpreted in the light of certain study limitations. First, because this was a retrospective study, the most substantive limitation is the absence of standardized criteria for diagnosing BED and comorbidities and the lack of standardized methods for assessing treatment outcomes. Second, the only clinically related exclusion criterion was a documented eating disorder. Because psychiatric and medical comorbidities are common among patients with BED [8, 10, 11], it is not known how comorbidities or the medications taken to treat these comorbidities might have influenced these findings. Third, detailed information on the treatment regimens (e.g., duration, frequency) and post-treatment follow-up visits could not be collected from patient charts, so conclusions regarding efficacy, time to improvement, and the nature of follow-up visits cannot be ascertained. Data on weight change were also not collected. Fourth, post-treatment data were not available for all patients. Fifth, response rate was low (57 participants from 4729 distinct invitations), and the sample sizes were small, so these data may not be representative of the general population of Canadian patients with BED. In addition, the HCPs included in this study had previously participated in BED-related research and may not be representative of the general community of HCPs. Sixth, the data are presented using descriptive statistics, so comparisons between GPs and psychiatrists and among therapies should be made cautiously. Despite the fact that these limitations constrain the substantive conclusions that can be drawn from this study, this study does provide valuable real-world data that highlight the patient journey from symptom onset to treatment.

In conclusion, the results from this study indicate that the journey for patients with BED is typified by HCPs initiating the discussion about BED, with GPs being the point of first consultation more frequently than psychiatrists. Most patients experienced symptoms of BED for > 1 year before being diagnosed with BED, suggesting that a more timely assessment and diagnosis of BED is required. Patients were treated with nonpharmacotherapy more often than pharmacotherapy, with all treatment options being associated with reduced BE and improved overall well-being. These findings suggest that multiple treatment options should be considered by HCPs, with decisions taking into account the characteristics of the patient and the treatment. Taken together, these findings highlight the importance of the timely diagnosis and treatment of patients with BED to make the patient journey from symptom onset to diagnosis and treatment less burdensome, thus potentially ensuring better outcomes.

### What is already known

Binge eating disorder (BED) is the most prevalent eating disorder. Multiple therapies are available, but understanding of the patient journey from symptom onset to treatment in Canada is poor.

### What this study adds

The BED patient journey is typified by HCPs initiating BED discussions, general practitioners being the initial consult, and most patients experiencing symptoms for > 1 year before being diagnosed.
